# Combining micromagnetism and magnetostatic Maxwell equations for multiscale magnetic simulations^☆^

**DOI:** 10.1016/j.jmmm.2013.04.085

**Published:** 2013-10

**Authors:** Florian Bruckner, Christoph Vogler, Bernhard Bergmair, Thomas Huber, Markus Fuger, Dieter Suess, Michael Feischl, Thomas Fuehrer, Marcus Page, Dirk Praetorius

**Affiliations:** a Vienna University of Technology, Institute of Solid State Physics, Austria; b Vienna University of Technology, Institute for Analysis and Scientific Computing, Austria

**Keywords:** LLG, Micromagnetism, Magnetostatic Maxwell equation, Multiscale, Time integration

## Abstract

Magnetostatic Maxwell equations and the Landau–Lifshitz–Gilbert (LLG) equation are combined to a multiscale method, which allows to extend the problem size of traditional micromagnetic simulations. By means of magnetostatic Maxwell equations macroscopic regions can be handled in an averaged and stationary sense, whereas the LLG allows to accurately describe domain formation as well as magnetization dynamics in some microscopic subregions. The two regions are coupled by means of their strayfield and the combined system is solved by an optimized time integration scheme.

## Highlights

• Coupling LLG and magnetostatic Maxwell equations.
• Optimized time-integration.
• Simulation of transfer curve of GMR read head with macroscopic shields.

## 1 Introduction

Micromagnetic simulations are utilized in a wide range of applications ranging from magnetic storage devices, permanent magnets to spintronic devices. With increasing complexity of the devices more properties have to be included in the simulations in order to predict the functional behavior of the structures accurately. State of the art micromagnetic simulations can handle systems with several millions of unknowns. In order to tackle these large scale problems both (i) new hardware architectures [1,2] as well as (ii) advanced numerical methods are required.

Newly developed numerical methods focus on speeding up the two most time consuming parts in micromagnetic simulations, which are the calculation of the strayfield and the time integration of the LLG equation. Advanced time integrations schemes can be found in Refs. [3–8]. For the calculation of the strayfield advanced FFT algorithms [9,10], fast multipole methods [11,12], nonuniform grid methods [13], FEM/BEM coupling approaches including compression of the boundary matrix [14–16], and tensor grid methods [17,18] have been developed.

Aside from new algorithms solving the micromagnetic model efficiently for systems with many degrees of freedom, it is often possible to choose a simplified physical model to describe at least some parts of the total problem. By this way the number of degrees of freedom can be reduced dramatically without loosing accuracy in regions where it is desired. Within this paper we will utilize the fact that models described by the LLG equation require very fine grained discretization which can lead to impractically large system sizes. We propose using the LLG equation to describe only those regions of the problem where detailed information about the domain structure such as domain walls and vortex structures are required. For the rest of the model a macroscopic description via magnetostatic Maxwell equations is chosen. Since it does not resolve the detailed domain structure it allows to use much coarser discretization.

In contrast to the multiscale method presented in this paper there exist methods which solve combined LLG–Maxwell equations within the whole problem region [19–21]. These methods extend the ordinary LLG model, by allowing to describe eddy currents or other dynamic effects, but they do not address the discretization size constraint and are therefore not suitable for large scale problems.

The structure of the paper is as follows. Section 2 summarizes the methods that are used to individually solve the LLG equations or magnetostatic Maxwell equations respectively. How the two systems can be coupled in an efficient way is described in Section 3. Finally in Section 4 the multiscale algorithm is applied to the simulation of a magnetic giant magnetoresistance (GMR) read head and numerical results and benchmarks are presented.

## 2 Fundamentals

**Fig. 1:**
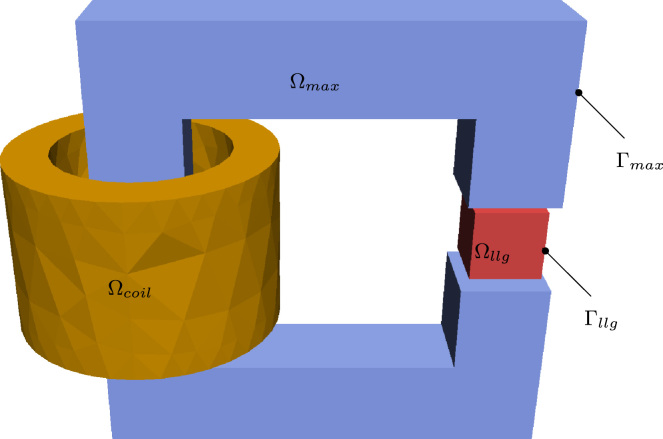
Example geometry which demonstrates model separation into LLG region $\Omega_{\mathrm{llg}}$ and Maxwell region $\Omega_{\max}$ (and in this case in an electric coil region $\Omega_{\mathrm{coil}}$). The boundaries of the regions are called $\Gamma_{\mathrm{llg}}$ and $\Gamma_{\max}$ respectively.

For the coupling of micromagnetism and magnetostatic Maxwell equations the full model is divided into two separated regions (see Fig. 1). The LLG equation is used to describe the first region $\Omega_{\mathrm{llg}}$, where domain structure, short range interactions or the magnetization dynamics of the magnetic parts is of great interest. The second region $\Omega_{\max}$ is described by magnetostatic Maxwell equations, which describe the magnetic state in a spatially averaged sense and without dynamics. Since both models contain the external field as a source term, coupling via the strayfield can be achieved in a straightforward way. The strayfield created from the LLG model can be considered as an external field of the Maxwell model and vice versa. An additional region $\Omega_{\mathrm{coil}}$ allows to define currents in a nonmagnetic medium, which in turn creates the source field for the magnetic model. The solution of the open-boundary problem requires the definition of the boundaries of the LLG-region ($\Gamma_{\mathrm{llg}}$) as well as of the Maxwell region ($\Gamma_{\max}$). In the following subsections it is shown how the two sub-problems are solved individually.

### 2.1 LLG

The LLG equation describes how magnetic polarizations J (with a fixed modulus *J*_*s*_) evolve in an effective field $H_{\mathrm{eff}}$. It consists of a precessional term as well as a phenomenological damping term

(1) $$\frac{\partial J}{\partial t}=-\frac{\left|\gamma \right|}{1+\alpha^{2}}J\times H_{\mathrm{eff}}-\frac{\alpha }{1+\alpha^{2}}\frac{\left|\gamma \right|}{J_{s}}J\times J\times H_{\mathrm{eff}}$$

where α is the Gilbert damping constant, *J*_*s*_ is the saturation polarization and $|\gamma |=\mu_{0}|\gamma_{e}|=2.210175\times 10^{5}\;m/\mathrm{As}$ is the reduced gyromagnetic ratio (with $\mu_{0}$ the permeability of the free space and $\gamma_{e}$ the gyromagnetic ratio of the electron). The effective field can be split into four contributions as follows:

(2) $$H_{\mathrm{eff}}=H_{\mathrm{ex}}+H_{\mathrm{ani}}+H_{\mathrm{demag}}+H_{\mathrm{ext}}=\frac{2A}{J_{s}^{2}}\Delta J-\frac{2}{J_{s}^{2}}K_{1}(J\cdot a)a+H_{\mathrm{demag}}+H_{\mathrm{ext}}$$

$H_{\mathrm{ex}}$ describes the short-range exchange interaction parametrized by the exchange constant *A*. $H_{\mathrm{ani}}$ stands for the magneto-crystalline anisotropy field with the uniaxial anisotropy constant *K*_1_ and the easy axis a. The magnetic strayfield $H_{\mathrm{demag}}$ describes the long-range interaction between the magnetic moments within the magnetic medium. $H_{\mathrm{ext}}$ is the applied field, which can for example be created by an electric coil, or as described later on by a Maxwell model. In addition to the mentioned fields several other contributions are possible, like terms taking into account thermal fluctuations or magneto-elastic interactions.

To calculate the strayfield created by a given magnetization distribution, which is needed for $H_{\mathrm{demag}}$ and also for the interaction between LLG and Maxwell parts, the Fredkin–Koehler method [14] is used. Basically the following equations for the scalar potential *u*_*llg*_ are solved for given J:

(3a) $$\nabla^{2}u_{\mathrm{llg}}=\nabla \cdot J\;\mathrm{in}\;\Omega_{\mathrm{llg}}$$

(3b) $$\nabla^{2}u_{\mathrm{llg}}=0\;\mathrm{in}\;R^{3}â§¹\Omega_{\mathrm{llg}}$$

(3c) $$[u_{\mathrm{llg}}]=0\;\mathrm{on}\;\Gamma_{\mathrm{llg}}$$

(3d) $$[\frac{\partial u_{\mathrm{llg}}}{\partial n}]=n\cdot J\;\mathrm{on}\;\Gamma_{\mathrm{llg}}$$

where [x] means the jump of value *x* at the surface of the LLG region. The strayfield finally reads as $H_{\mathrm{demag}}=-\mu_{0}^{-1}\nabla u_{\mathrm{llg}}$.

A detailed description of how the LLG equation is actually solved as well as a proper preconditioning method to speed up calculations of large problems can be found in [3].

### 2.2 Magnetostatic Maxwell equations

Magnetostatic Maxwell equations are used to describe stationary phenomena averaged over a sufficiently large area. As a result of this description the modulus of the magnetic polarizations is not constant, but depends on the local magnetic field. Since the material behavior is only described in average, its description leads to a more complicated (in general nonlinear) material law. The advantage of this approach is that it allows to discretize the geometry much coarser, because domains need not be resolved, and that it allows to ignore the possibly disturbing dynamics of the macroscopic parts.

The fundamental equations that have to be solved within the Maxwell region are

(4a) rotH=jdivB=0

(4b) $$B=\mu_{0}(H+M)=\mu_{0}\mu H$$

where the current density j is the source of the magnetic field strength H which is related to the magnetic flux density B via the relative permeability μ (which may depend on the location and in the nonlinear case also on the local field strength) times the vacuum permeability $\mu_{0}=4\pi 10^{-7}\;\mathrm{Vs}/\mathrm{Am}$. In contrast to the strayfield calculation of the LLG model the magnetization M is not known a priori since it depends on the local magnetic field strength.

Introduction of a reduced scalar potential *u*_*max*_ by setting $H=H_{\mathrm{ext}}-\nabla u_{\max}$ directly solves the homogeneous Maxwell equation and combined with proper jump condition at the boundary of the magnetic parts it leads to

(5a) $$\nabla \cdot (\mu \nabla u_{\max})=\nabla \cdot (\mu H_{\mathrm{ext}})\;\mathrm{in}\;\Omega_{\max}$$

(5b) $$\nabla^{2}u_{\max}=0\;\mathrm{in}\;R^{3}â§¹\Omega_{\max}$$

(5c) $$[u_{\max}]=0\;\mathrm{on}\;\Gamma_{\max}$$

(5d) $$[\mu \frac{\partial u_{\max}}{\partial n}]=[\mu ]n\cdot H_{\mathrm{ext}}\;\mathrm{on}\;\Gamma_{\max}$$

where [x] means the jump of value *x* at the surface of the Maxwell region.

A detailed description of the methods used to solve the magnetostatic Maxwell equations can be found in [22].

### 2.3 Discretization

The inhomogeneities within the LLG- as well as within the Maxwell-domain are discretized by means of finite elements. Within the LLG domain the element size is constrained by the exchange length of the used material. Typical values are in the range of 10 nm. Choosing larger elements would lead to unphysically large domain wall widths. For the Maxwell region such constraint does not exist, which allows to use much larger elements in some regions.

In both cases FEM–BEM coupling methods are applied to handle the open-boundary problem. In addition to the fact that these methods are well suited for the solution of the individual problems they also simplify the coupling of the two methods because each methods can be solved on its individual mesh without the need for a global mesh. The strayfield produced by each model which is needed to handle interactions can be calculated at any point by means of the boundary element formulas.

## 3 Coupling method

In order to solve the coupled problem one needs to deal with ordinary differential equations (ODE), which arise from the spatial discretization of the LLG equations, as well as with algebraic equations arising from Maxwell's equations. Discretization of this system of Differential-Algebraic-Equations (DAE) using integration methods for ODEs can lead to numerical instabilities or to a drift error in the algebraic equations [23]. Therefore differential and algebraic equations are kept separate and a sequential method is used to combine both problems. A first implementation simply solves the Maxwell problem every time the right-hand side of the LLG equation is solved. In an abstract notation this can be written as

(6) $${\overset{˙}{y}}_{\mathrm{llg}}=\mathrm{LLG}(t,y_{\mathrm{llg}},y_{\max}=\mathrm{MAX}(t,y_{\mathrm{llg}}))$$

where *y*_*llg*_ and *y*_*max*_ are the unknowns of the LLG as well as of the Maxwell models. For time discretization the backward differential formula (BDF) is applied to the ODE system and is in turn solved by means of an Inexact Newton method (we therefore used the open source differential equation solver CVODE [24]). Since an implicit time integration scheme is used, which needs to approximately solve a system of equations within every timestep, Eq. (1) needs to be evaluated several times during each timestep. In order to calculate *H*_*ext*_ within the right-hand side of the LLG equation, the Maxwell problem (5) is solved under consideration of the strayfield produced by the actual magnetization of the LLG model. After the Maxwell system is solved the back-interaction on the LLG model can be calculated and allows to finally evaluate the right-hand side of the LLG system. This procedure leads to a fully implicit scheme to solve the coupled equation.

### 3.1 Optimization

Solving the Maxwell problem within every function evaluation of the LLG time-integration can be very time-consuming for considerably large Maxwell models. Fortunately the simulation can be speeded up by using the theoretical prediction [25] that for stability of the time-integration only the exchange interaction term needs to be handled implicitly. All other terms, including the interaction with the Maxwell model, can be handled explicitly, which means that we simply use the interaction field of the last timestep to evaluate all function values needed at the next timestep. Thus we only need to solve the Maxwell problem once for every nonlinear step of the time-integration.

## 4 Results

### 4.1 Problem description

**Fig. 2:**
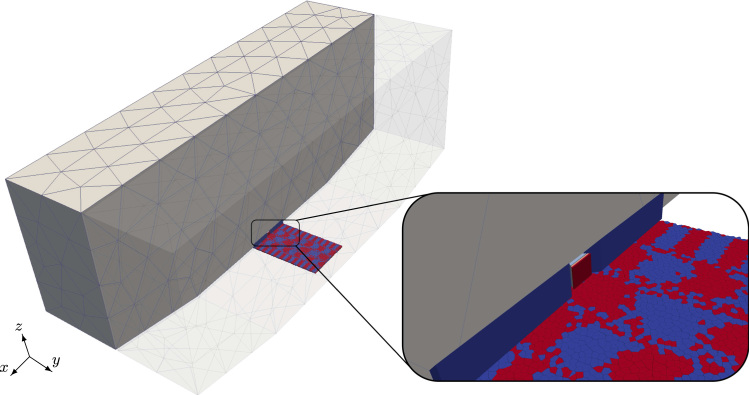
The example setup consists of a GMR sensor element in between two macroscopic shields (5μm×2μm×2μm). Beyond the GMR sensor a magnetic storage medium is indicated (it will not be considered for the calculation of the transfer curves).

In this section the developed algorithm is applied to calculate the transfer curve [26] of a magnetic read head setup (see Fig. 2). For the transfer curve a homogeneous external field perpendicular to the medium is applied to the whole setup and the stationary output of the read head is plotted as a function of the field strength. The medium is not considered in this simulation. In practice such transfer curves are used to characterize magnetic read heads and they are experimentally measured with field sweep rates much lower than the rates which occur in the actual magnetic recording process.

The (GMR) reader element consists of microscopic layers which can be well described by the LLG. On the other hand the setup consists of macroscopic shields which have the purpose to reduce the influence of the strayfield from neighboring bits on the output signal of the current bit. Since these shields are separated from the sensor element and its domain structure is not of interest, it is possible to describe them using magnetostatic Maxwell equations. Additionally for the transfer curve stationary states for different values of the external field are needed, which means that the dynamic of the shields can be safely ignored in this case. Therefore the usage of the multiscale method allows to reduce the simulation time significantly.

**Fig. 3:**
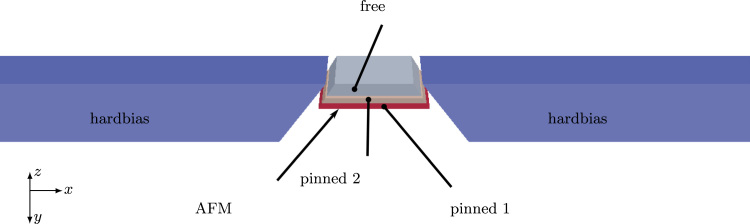
The GMR reader element for our test case consists of two pinned layers which are antiferromagnetically coupled via a thin ruthenium layer between them. Their magnetization is pinned by a granular antiferromagnetic layer below the bottom layer. The initial magnetization of these two layers is chosen to direct in-plane into the *z* or −z direction respectively. The freelayer is located above the pinned layers and its initial magnetization is forced orthogonal to the magnetization of the pinned layer by means of two hardbias magnets whose easy axis shows into the *x*-direction. The output signal of the sensor element is proportional to the cosine of angle ϕ between the magnetization within the pinned- and the free-layer. For sake of simplicity electrodes are not included in this model. The total size of the model is 550 nm×60 nm×20 nm.

The detailed structure of the giant magnetoresistance sensor is shown in Fig. 3. Due to the GMR effect the resistance of the freelayer changes depending on the cosine of angle ϕ between the magnetization within free- and pinned layer. The transfer curve thus shows cos(ϕ) for various external field strengths.

**Fig. 4:**
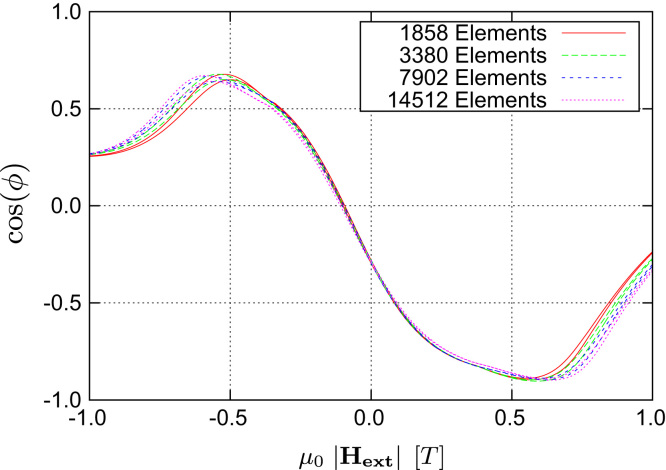
Calculated transfer curves of the presented multiscale algorithm for various shield grid sizes. The angle ϕ between the magnetizations within pinned- and free-layer is plotted as a function of the external field $H_{\mathrm{ext}}$. The algorithms starts to produce convergent results at around 1000 elements and due to the use of magnetostatic equations it shows no dynamic artifacts.

In order to demonstrate the strengths of the presented algorithm we calculate transfer curves for different grid sizes of the magnetic shields. The results of the multiscale algorithm (see Fig. 4) are compared with those of an LLG-only simulation. For the material of the Maxwell part we use a linear material law with permeability μ=1000 up to the saturation polarization *J*_*s*_, which is the same as in the LLG-only case. The LLG parameters used are summarized in Table 1. Because we are only interested in the stationary state for certain external field amplitudes, α=1 has been chosen, which maximizes the energy dissipation and therefor leads to the fastest transition into the stationary state. A complete hysteresis is calculated by changing the applied field amplitude from −2T to +2T and then back to −2T again. By this way it is possible to check the reproducibility of the stationary states.

### 4.2 Simulation results

**Fig. 5:**
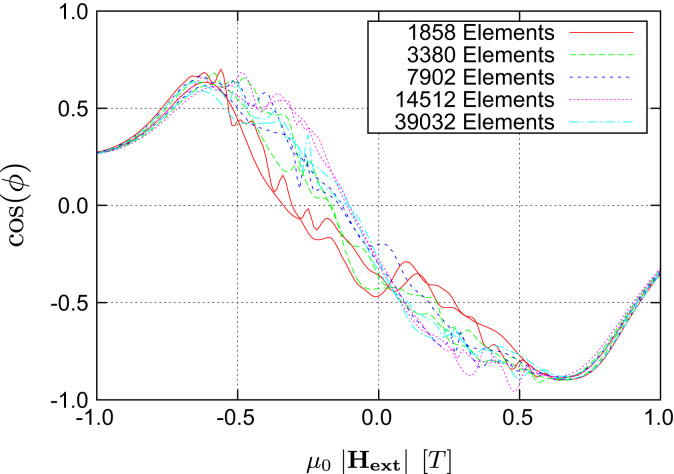
Calculated transfer curves of LLG-only simulations for various shield grid sizes. In contrast to the multiscale algorithm it shows some significant deviations up to at least 50,000 elements. Additionally there occur some artifacts due to the large time constant of the magnetization dynamic within the macroscopic shields. The field sweep rate is chosen too high to reach the equilibrium state and therefore leads to some fluctuations within the transfer curves.

**Fig. 6:**
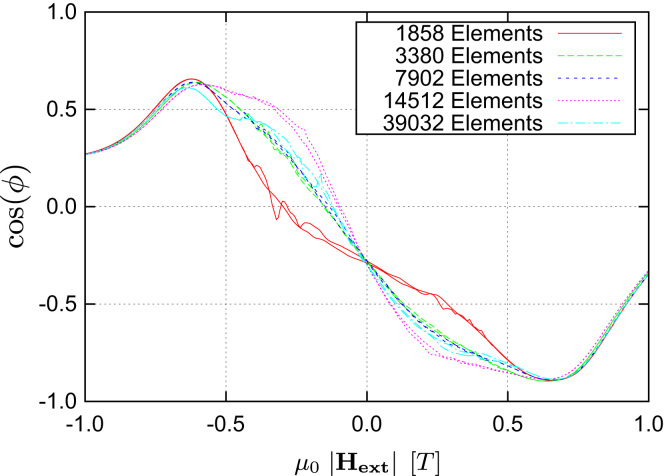
Calculated transfer curves of LLG-only simulations using a field sweep rate reduced by a factor 5. The fluctuations are reduced, but there are still significant differences due to the coarse shield discretization.

**Fig. 7:**
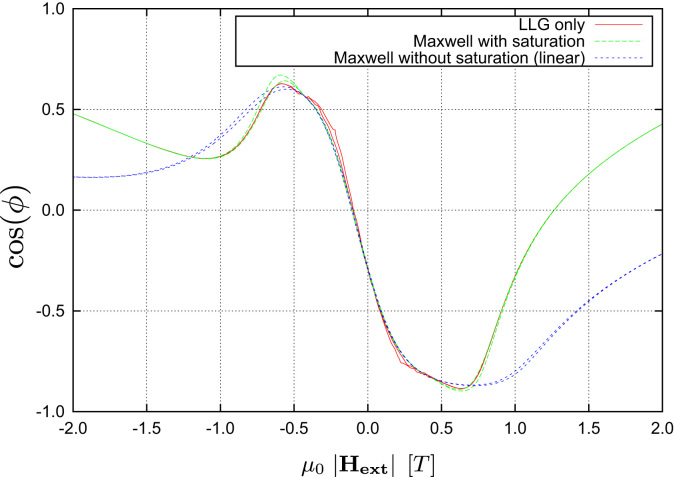
Simulation results of the multiscale solver compared with an LLG-only calculation. Both algorithms are applied to the same model with 14,512 elements. A permeability of $10^{3}$ is used for the material of the shields within the Maxwell solver. In order to get matching results at high fields one needs to use a saturated material law.

Starting with an LLG-only simulation using an external field sweep rate of 0.2T/ns one can clearly see that the stationary states are not reached and the curves show strong dynamic fluctuations (see Fig. 5). Thus another LLG-only simulation with a much slower field sweep rate was performed and it was possible to significantly reduce the dynamic artifacts in the calculated transfer curves (see Fig. 6). Nevertheless one also notices that both versions of the LLG-only simulation suffer from a strong dependence on the grid size used for the discretization of the magnetic shields. Due to the use of magnetostatic Maxwell equations both of these problems do not occur when using the multiscale method (see Fig. 4). Finally a comparison of the different algorithms for proper grid sizes as well as field sweep rates (see Fig. 7) shows that the results are in good agreement with each other, provided that one uses a saturated material law within the Maxwell part.

A comparison of the performance of the multiscale algorithm with and without optimization, as well as with LLG-only methods is presented in Table 2.

## 5 Conclusion

A multiscale algorithm was presented which combines the capabilities of LLG- as well as Maxwell-equation solvers and allows to handle much larger problem sizes. Coupling the LLG to the Maxwell part could be optimized by handling the corresponding terms explicitly within the time integration scheme. Finally the optimized algorithm was validated by means of a transfer curve simulation of a magnetic read head. The results of the multiscale algorithm match very well with those of the LLG-only simulation, but it allows to significantly reduce the shield grid size and to increase the field sweep rates.

## Figures and Tables

**Table 1:** Summary of LLG parameters used for the read head simulation. The easy axis of all anisotropic materials is directed in *x*-direction.

	Uniaxial anisotropy constant *K*_1_ (J/m^3^)	Magnetic polarization *J*_*s*_ (T)	Exchange constant *A* (J/m)	Gilbert damping constant α [1]	Layer thickness *t* (m)
Shields	200.0	1.0	1.25e−11	1.0	–
Hardbias	1.0e−6	0.6	1.25e−11	1.0	–
Free	20.0	1.21	1.25e−11	1.0	–
Pinned 2	20.0	1.0	1.25e−11	1.0	–
Pinned 1	20.0	1.0	1.25e−11	1.0	–
AFM	0.0	0.01	−8.0e−13	1.0	0.8e−9

**Table 2:** Algorithm performance for the transfer curve calculation. *t*_*run*_ is the overall runtime of the simulation. The simulation period *t*_*sim*_ as well as the shield grid size *N*_*shield*_ are chosen in a way that the algorithm produces convergent results (LLG ${}^{⁎}$ shows non-converged results and is listed only to give a reference time scale). *r*_*sweep*_ is the sweep rate of the applied external field. *N*_*max*_ and *N*_*llg*_ are the number of times the Maxwell- or the LLG-part is evaluated, respectively.

	LLG^⁎^	LLG (conv.)	Multiscale	Multiscale (optimized)
*t*_*sim*_ (ns)	70	1000	70	70
*r*_*sweep*_ (T/ns)	0.2	0.01	0.2	0.2
*N*_*shield*_	3380	39,032	3380	3380

*N*_*max*_	–	–	22,274	6038
*N*_*llg*_	69,584	173,227	22,274	22,203

*t*_*run*_ (s)	184	173,184	11,857	8107
